# MTB-ImmunogenKG: An LLM-assisted knowledge graph for antigen selection in tuberculosis vaccine research

**DOI:** 10.1016/j.bsheal.2026.02.001

**Published:** 2026-02-04

**Authors:** Jielong Peng, Xinhao Zhuang, Yingying Chen, Haitong Xu, Yunjie Du, Bingdong Zhu, Guoping Zhao, Ying Wang, Yunchao Ling, Guoqing Zhang

**Affiliations:** a Bio-Med Big Data Center, Shanghai Institute of Nutrition and Health, University of Chinese Academy of Sciences, Chinese Academy of Sciences, Shanghai 200031, China; b Shanghai Institute of Immunology, Department of Microbiology and Immunology, Shanghai Jiao Tong University School of Medicine, Shanghai 200025, China; c School of Life Science, Hangzhou Institute for Advanced Study, University of Chinese Academy of Sciences, Hangzhou 310024, China; d State Key Laboratory for Animal Disease Control and Prevention, Lanzhou Center for Tuberculosis Research, Institute of Pathogen Biology, School of Basic Medical Sciences, Lanzhou University, Lanzhou 730000, China; e Shanghai Institute of Virology, Shanghai Jiao Tong University School of Medicine, Shanghai 200025, China

**Keywords:** *Mycobacterium tuberculosis* (MTB), Vaccine development, Antigen prioritization, Knowledge graph, Large language model (LLM), Literature mining

## Abstract

Tuberculosis (TB) vaccine design relies on the selection of optimal antigens. However, evidence on candidate antigens is scattered across the literature and rarely linked to critical vaccine parameters, such as immune responses, antigen combinations, and adjuvants. Here, we introduce *Mycobacterium tuberculosis* (MTB)-ImmunogenKG, a provenance-linked, antigen-centric knowledge graph for MTB, that is constructed *via* an optimized information-extraction pipeline and integrated with a large language model (LLM) system to enable knowledge-augmented reasoning. From over 77,000 publications indexed in PubMed (as of July 2024), this graph consolidates 1.48 million sentence-level statements spanning 14 entity types. In practical applications, MTB-ImmunogenKG enables contradiction-aware antigen profiling for 3,154 proteins (representing about 77% of the total annotated proteins) and improves protective-efficacy prediction with a Matthews correlation coefficient (MCC) gain of 0.19 over sequence-based tools and 0.45 over an LLM-only baseline. By delivering a traceable synthesis of fragmented findings, MTB-ImmunogenKG facilitates the construction of antigen panels and the pairing of adjuvants, thereby streamlining the experimental design cycle for next-generation TB vaccines.

## Highlights

• **Scientific questions**: Vaccine development constitutes a pivotal strategy to combat the major global health threat of tuberculosis (TB). However, identifying optimal antigens for these vaccines remains a formidable challenge due to the vast number of Mycobacterium tuberculosis (MTB) proteins.
• **Evidence before this study**: Information on TB antigens is currently fragmented across databases that require manual synthesis and lacks integration with key vaccine parameters. Furthermore, existing computational prediction tools rely primarily on sequence properties, failing to leverage the rich experimental immunological evidence scattered in the literature.
• **New findings**: We mined 77,704 papers to construct an antigen-centric knowledge graph (MTB-ImmunogenKG) consolidating 1.48 million sentence-level statements to profile 3,154 proteins. In protective-efficacy prediction, our KG-augmented large language model (LLM) classifier achieved a Matthews correlation coefficient (MCC) of 0.40, surpassing LLM-only baselines by 0.45 MCC points and sequence-based tools by 0.19.
• **Significance of the study**: This work establishes a publicly available, provenance-linked resource that streamlines the experimental design cycle by facilitating rational antigen prioritization and adjuvant pairing for next-generation vaccines.

## 1 Introduction

Tuberculosis (TB), caused primarily by *Mycobacterium tuberculosis* (MTB), remains a major global health threat, with 10.8 million new cases and an estimated 1.25 million deaths in 2023 [1], [2], [3]. The century-old Bacillus Calmette–Guérin (BCG) vaccine provides inconsistent protection, particularly against adult pulmonary disease [4], [5], [6], [7], [8], underscoring the need for new vaccines [9]. TB vaccine protection is primarily T-cell-mediated and depends on human leukocyte antigen (HLA) molecules presenting antigen-derived epitopes for T-cell recognition [10], [11]. Accordingly, the identification of antigens that elicit protective T-cell responses is central to vaccine design yet remains challenging [12], [13], [14], [15].

Rational antigen selection is a decision problem defined by explicit criteria [16], [17], [18]. However, the evidence supporting these criteria is dispersed across narrative text that intertwines antigens with immune context [19], impeding the derivation of auditable rationales and decision support. Existing computational approaches address fragments of the problem, but do not bridge the gap between evidence and decision. Specialized databases increase findability yet still require manual synthesis across heterogeneous sources. Representative resources include the tuberculosis database (TBDB), COMBAT-TB-NeoDB, AntiTbPdb, and immune epitope database (IEDB) [20], [21], [22], [23]. Large language models (LLMs) can extract relevant evidence at scale from the biomedical literature, but their reliability for decision support is limited by factual inaccuracies and hallucinations [24]. As a result, structured data in databases remains weakly coupled to actionable decisions, whereas unstructured knowledge surfaced by LLMs is often unreliable.

We introduce MTB-ImmunogenKG, an antigen-focused knowledge graph designed to connect evidence with decision-making through natural-language access and provenance-linked profiles. The resource integrates 1.48 million evidence instances from more than 77,000 publications. It was built with Cavistill, a distillation-based biomedical information extraction framework that assigns sentences to four knowledge patterns—Antigen, Host immune response, Antigen synergy, and Adjuvant, and performs named entity recognition (NER). This work details the graph, the extraction method, and its application to antigen assessment. By reframing rational antigen selection as a large-scale knowledge engineering task, the study delivers transparent and auditable decision support grounded in a comprehensive evidence base.

## 2 Materials and methods

### 2.1 Literature corpus and knowledge pattern

**Fig. 1:**
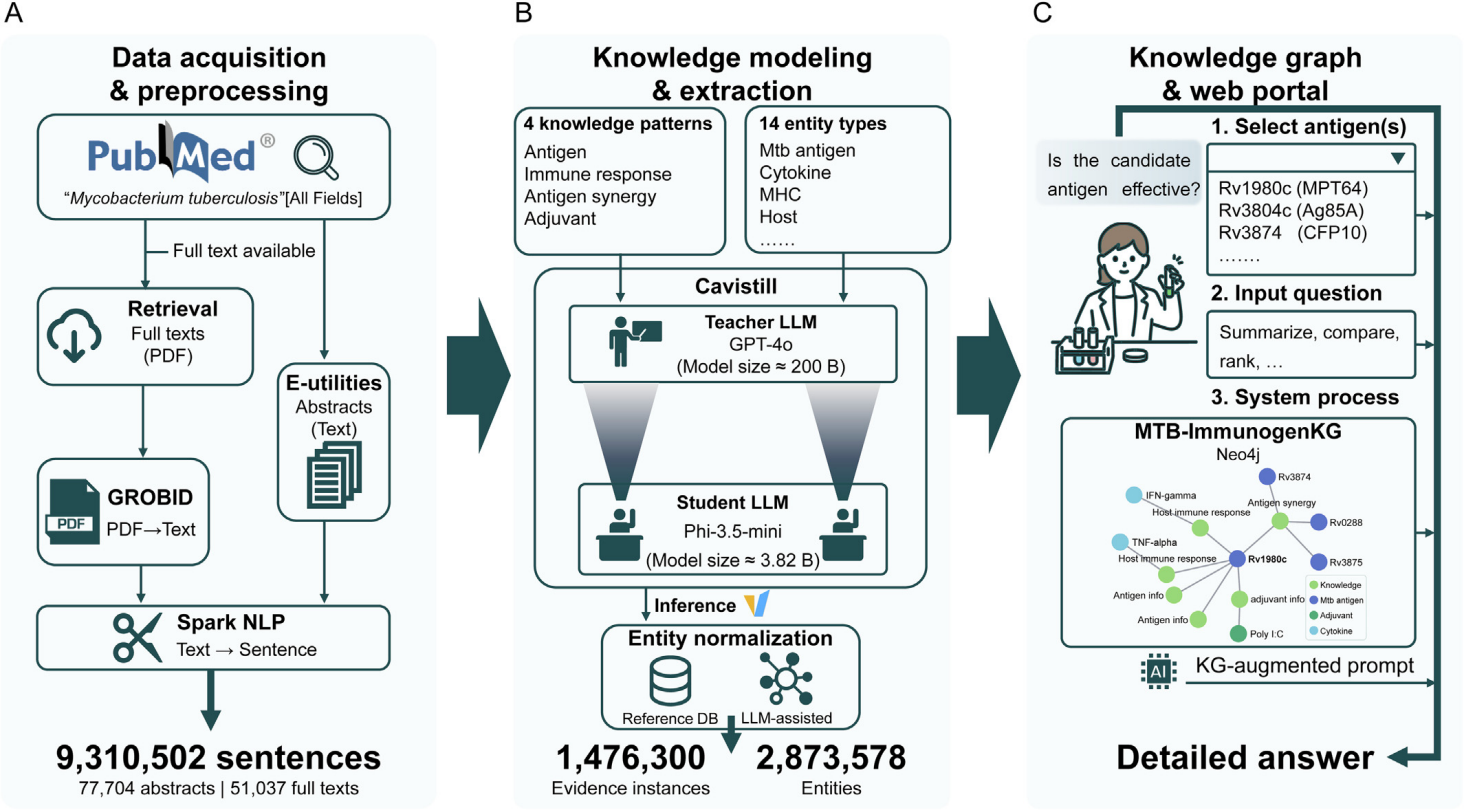
Construction of MTB-ImmunogenKG. A) Data acquisition & preprocessing. PubMed corpus of MTB literature was converted to plain text and segmented into sentences. B) Knowledge modeling & extraction. Four vaccine immunology knowledge patterns guide sentence-level classification and named entity recognition using the Cavistill distillation framework, followed by entity standardization. C) Knowledge graph & web portal. Evidence and entities are ingested into a Neo4j graph to support exploration; the web portal provides knowledge-graph-augmented, detailed answers based on the selected antigens and the input question. Abbreviations: MTB/Mtb, *Mycobacterium tuberculosis*; MHC, major histocompatibility complex; NLP, natural language processing; LLM, large language model; B, billion.

An unrestricted PubMed search for “*Mycobacterium tuberculosis*” [all fields] returned 77,704 records as of July 2024. Of these, we retrieved full-text PDFs for 51,037. For the remaining records, we retrieved abstracts as plain text using Entrez E-utilities [25]. As our downstream tasks rely on LLMs that operate on plain text and show degraded performance with long inputs [26], [27], we converted full-text PDFs to plain text with GROBID [28]. We then segmented all text into sentences with Spark natural language processing (NLP)’s SentenceDetectorDLModel [29], yielding 9,310,502 short sentences (median 29 tokens; Fig. 1).

To analyze this corpus, we introduce knowledge patterns, a methodology successfully employed in our prior work [30], defined as task-aligned semantic templates designed to describe a specific type of knowledge. Each pattern characterizes a thematic scope by outlining the key entities and their context. In this study, knowledge patterns are designed to capture the key evidence needed for TB antigen selection. This selection process is challenging due to the vast number of protein candidates in MTB [31], [32], [33], making evidence about the Antigen itself essential. Beyond the candidate, effective selection depends on assessing the Host immune responses elicited, as this indicates potentiated anti-TB immunity [11], [34]. Moreover, the prevalence of latent TB necessitates a search for Antigen synergy, identifying protein combinations that are effective across different stages of infection [35], [36]. Finally, because T-cells are central to anti-TB immunity, choosing Adjuvants that can enhance T-cell responses is crucial for successful subunit vaccine development [37], [38].

Based on these selection criteria, four patterns are used: Antigen, Host immune response, Antigen synergy, and Adjuvant. Formal definitions, comprehensive entity lists, and illustrative examples for each pattern are provided in Table 1. These patterns allow us to filter the literature to sentences that are directly informative for antigen assessment and to guide entity extraction and linkage.

**Table 1:** Knowledge patterns and statistics of MTB-ImmunogenKG.

Knowledge pattern and definition	Entity types	Source articles, n	Evidence instances, n	Entity mentions, n	Instances per article	Entities per instance
**Antigen**: Sentences that describe the intrinsic properties, biological characteristics, or functional attributes of a MTB antigen.	Ag, H, St, Sec, VSC	31,464	330,079	557,639	10.49	1.69
**Host immune response**: Sentences that describe host immune responses, including MTB-triggered innate immunity, as well as MTB antigen-specific T-cell responses or B-cell responses.	Ag, CT, H, Ck, R, St, IR, Ab, Chk, C, MHC	42,942	1,243,726	2,585,006	28.96	2.08
**Antigen synergy**: Sentences that describe combinatorial usage of multiple MTB antigens and their synergistic, complementary effects.	Ag, CT, H, Ck, R, Sec, Chk, Adj, MHC, VSC	3,553	10,292	22,986	2.90	2.23
**Adjuvant**: Sentences that describe adjuvant usage, formulation, mechanisms, or their modulatory effects on antigen-specific immunity.	Ag, CT, H, Ck, R, IR, Adj, VSC	3,588	16,703	46,141	4.66	2.76
Totals		50,005	1,476,300	2,873,578	29.52	1.95

Notes: Totals are deduplicated counts across all four knowledge patterns. Corpus source is PubMed (details in Methods). Abbreviations: MTB, *Mycobacterium tuberculosis*; CT, cell type/subtype; H, host; St, strain; Sec, bacterial surface secretion system; MHC, major histocompatibility complex; VSC, vaccine structural component; Ck, cytokine; R, receptor; IR, type of immune response; Ab, antibody; Chk, chemokine; Adj, adjuvant; C, complement; Ag, antigen.

### 2.2 Knowledge extraction

Directly applying a high-capacity language model to extract knowledge from the full corpus yields strong accuracy but is prohibitively expensive in compute and runtime at corpus scale [39], [40], [41], [42], [43], [44], [45]. To retain performance while reducing cost and run-to-run variance, we adopt knowledge distillation [46], [47]. A teacher produces calibrated supervision, which we use to fine-tune a compact student for two tasks—knowledge pattern classification and named entity recognition. In our setup, the teacher is GPT-4o [48] and the student is phi-3.5-mini-instruct (about 3.82 billion parameters) [49]. Cavistill standardizes prompting and performs multi-pass teacher inference with light randomization. It aggregates outputs into confidence-weighted labels, calibrates accept and reject cases, and then fine-tunes the student. The distilled model is used to process the entire corpus.

For the first task, knowledge pattern classification, we used a seed-and-iterate procedure to build supervision for pattern classification. A small set of seed sentences per pattern was manually annotated as exemplars in the teacher prompt; batches of 1,000 sentences were iteratively sampled from the full sentence corpus, labeled by the teacher, de-duplicated, and monitored for class balance to create a final multi-label training dataset. We split the data 80/10/10 for train, validation, and test. We fine-tuned the student, then applied the resulting model with virtual LLM [50] to all sentences, retaining 1,476,300 sentences assigned to at least one pattern (Table 1).

Subsequently, the named entity recognition task targeted 14 entity types relevant to the patterns (for example, MTB antigen, cytokine, host; Table S1). Following the same procedure, the model was then applied to the pattern-retained sentences to extract entities. The extracted entities provide the nodes for inter-sentence linkage in the knowledge graph (Section 2.3).

### 2.3 Entity standardization

NER produced 14 entity types, but raw mentions contained synonyms, abbreviations, and spelling or naming variants that impeded linking and querying. To obtain a consistent representation within each type, we applied two complementary strategies: reference database mapping and LLM-assisted categorization.

When an authoritative resource existed, we mapped mentions to reference databases and then expanded coverage with curated synonyms and variants. In practice, MTB antigens were mapped to UniProt for H37Rv, adjuvants to AdjuvareDB, and immune molecules to UniProt for *Mus musculus* [51], [52].

Where no comprehensive database was available, we employed LLM-assisted categorization, which was divided into classification and clustering. For small, well-delimited sets (i.e., immune response types), an LLM performed classification by assigning mentions to one of four predefined classes: humoral, cell-mediated, mucosal, or innate. For categories that were broader and harder to delineate (for example, bacterial surface secretion systems and vaccine structural components), LLM-assisted text clustering proposed corpus-derived category labels and assigned mentions; ambiguous cases were left unmapped. Full mappings and coverage statistics are provided in Table S1.

### 2.4 Knowledge graph construction and antigen profiling workflow

We implemented MTB-ImmunogenKG in Neo4j (v5.19.0) to preserve sentence-level provenance and to support fast retrieval by entity. The graph has three node types: Paper, Sentence, and Entity. Each Sentence node links to its source Paper, preserving citation and context, and connects to Entity nodes.

**Fig. 2:**
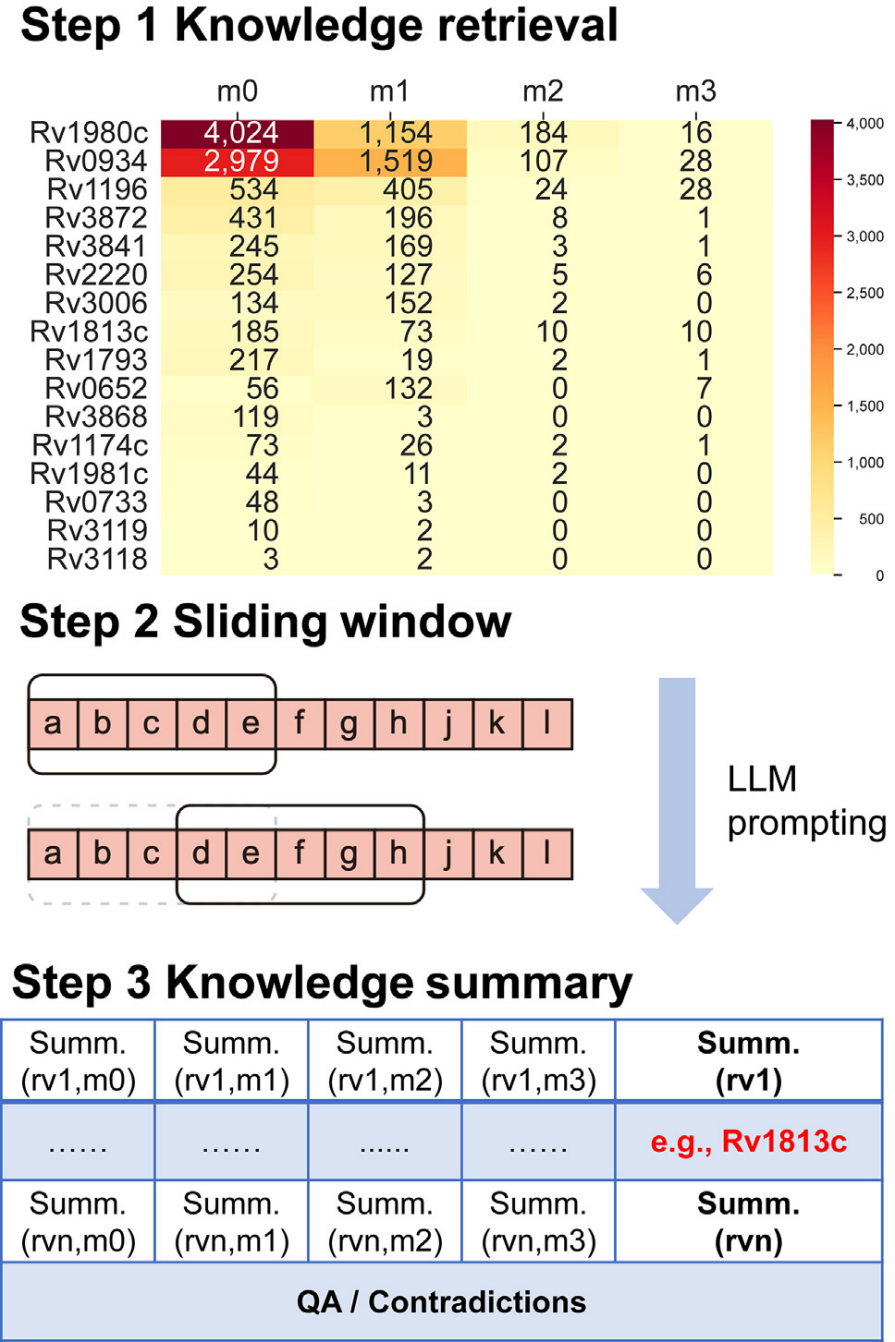
Three-stage knowledge-driven antigen profiling. Step 1 involves knowledge retrieval, where evidence is retrieved from the knowledge graph and grouped by four knowledge patterns: m0 (antigen), m1 (host immune response), m2 (antigen synergy), and m3 (adjuvant). Step 2 applies a sliding window approach to the retrieved sentences for antigens with extensive evidence to produce intermediate, pattern-level summaries. Step 3 generates a knowledge summary by consolidating the intermediate summaries into a final output, which synthesizes an overall answer and identifies potential contradictions from the evidence. In Step 3, rv1, ..., rvn denote arbitrary antigens in the profiling set, and each cell represents the corresponding pattern-level summary for a given antigen. Abbreviations: LLM, large language model; Summ., summary; QA, question answering.

Leveraging the constructed graph, we developed a workflow for knowledge-driven antigen profiling (Fig. 2). The workflow begins with Knowledge retrieval (Step 1), where queries retrieve all associated sentences with corresponding bibliographic information, grouped by the four knowledge patterns. This retrieved evidence is then synthesized: for antigens with extensive evidence, a Sliding window (Step 2) approach is applied, which first produces pattern-level summaries. Finally, these intermediate summaries are consolidated into a comprehensive Knowledge dummary (Step 3), which uses prompts to instruct the LLM to summarize the evidence and identify potential contradictions. The generated summaries and identified contradictions are displayed in a unified web interface, presenting the related topic, contrasting viewpoints, and supporting references for each contradiction to facilitate decision-making.

### 2.5 Prediction of antigen protective efficacy

To quantitatively evaluate the utility of MTB-ImmunogenKG, we designed an experiment to assess its contribution to predicting antigen protective efficacy using an independent evaluation set. Our evaluation strategy is a comparison between three approaches: (1) a KG-augmented model (GPT-4o provided with our knowledge summaries), (2) a Name-only control (GPT-4o provided with only the antigen name), and (3) traditional sequence-based bioinformatics tools. Performance for all approaches was assessed on a binary classification task to identify antigens with high protective efficacy. All metrics were computed on the entire evaluation set. We report the Matthews correlation coefficient (MCC) as an overall summary metric, together with precision, recall, and F1 score. We also analyzed per-antigen outcomes by categorizing classification changes as KG fix (where the KG-augmented model corrected a Name-only error) and KG break (where augmentation introduced a new error). Finally, we performed an error analysis on the KG-augmented model's most confident false positives. Complete per-antigen results are presented in Supplementary data.

The evaluation set consists of 42 MTB antigens tested in an *in vivo* study using CB6F1 mice challenged with H37Rv [53]. In this dataset, protection was measured as the fold reduction in lung colony forming unit (CFU). Following the source study, antigens with greater than 1.5-fold reduction were labeled Positive (indicating high protection). Antigens with 1.5-fold reduction or less were labeled Negative.

For the KG-augmented approach, evidence was retrieved from MTB-ImmunogenKG under the Antigen and Host immune response patterns. Relevant information was found for 40 antigens and synthesized into a condensed knowledge summary for each (two antigens were not covered by the graph; details in Supplementary data). For the Name-only control, only the antigen name was used. Predictions for both LLM conditions were generated with GPT-4o (prompts and predictive cues are reported in Supplementary data) [53], [54].

For the Seq-based comparison, we used Vaxign-ML [55], Vaxijen2 [56], and Vaxijen3 [57] to predict efficacy from the UniProt protein sequences of the 42 antigens.

## 3 Results

### 3.1 Construction of MTB-ImmunogenKG and summary statistics

We constructed MTB-ImmunogenKG with a multi-stage literature-mining pipeline (Fig. 1; Sections 2.1–2.2). Articles were converted to plain text and segmented into sentences, then processed by knowledge-pattern filtering and extraction. The resulting resource consolidates a large body of MTB literature into sentence-level, provenance-linked evidence and normalized entity mentions (Table 1). Notably, our knowledge graph identifies 3,154 unique MTB proteins as entities (Table S1). This represents 77% coverage of the 4,083 total annotated proteins reported for the organism (UniProt taxonomy ID 83332, accessed 08-Nov-2025). Indeed, by defining evidence at the sentence level, our knowledge pattern filtering markedly concentrates the evidence. This process reduces the average full-text article, with its approximately 150 total sentences [58], down to 29.52 high-relevance evidence instances per article.

Across the four knowledge patterns, the host immune response provides the largest volume of information. Antigen evidence is substantial but less dense. Content that is most directly relevant to vaccine potency, namely Antigen synergy and Adjuvant, appears in fewer articles. Each evidence instance tends to involve a broader set of distinct entities, reflecting greater compositional complexity; continued research in these areas is therefore needed. To place MTB-ImmunogenKG within the TB data landscape, Table 2 compares its capabilities with representative resources. It highlights broader literature coverage, native support for contradiction-aware evidence surfacing, and more comprehensive knowledge coverage that connects data retrieval to decision support for rational antigen selection.

**Table 2:** Comparison of MTB-ImmunogenKG with other TB-related databases.

Variables	MTB-ImmunogenKG	IEDB (MTB subset)	TBDB	COMBAT-TB-NeoDB	AntiTbPdb
Literature coverage	50,005 articles (curated from 77,704 articles)	659 articles	−	−	96 articles (curated from 10,652 articles)
Contradiction exposure	√	×	×	×	×
Antigen info	√	√	√	√	√
Host immune-response info	√	√	×	×	×
Cytokine info	√	√	×	×	×
Adjuvant info	√	×	×	×	×
Antigen-synergy info	√	×	×	×	×

“√” = feature fully supported; “×” = feature not supported; “–” = not applicable or data not available. Abbreviations: TB, tuberculosis; MTB, *Mycobacterium tuberculosis*; TBDB, tuberculosis database; info, information.

### 3.2 Knowledge-driven antigen profiling

We applied the knowledge-driven profiling workflow, detailed in Section 2.4, to 17 antigens, retrieving their evidence under the four knowledge patterns (Fig. 2). Information was concentrated in Antigen and Host immune response. Only a small subset showed broad coverage across all four patterns, consistent with prior concerns that current vaccine candidates rely on narrow antigen representation and immune profiles [14].

The resulting contradiction-aware summaries (Supplementary data) surfaced contradictions. For example, Rv1813c shows potential conflicting evidence regarding host immune response. One study [59] mentions that Rv1813c immunization elicits a proliferative recall response with cytokine induction, whereas another study [60] mentions that the Rv1813c deoxyribonucleic acid (DNA) vaccine does not increase type 1 helper T cells (Th1) or type 1 cytotoxic T cells (Tc1) proportions, indicating immune tolerance. Crucially, by clearly presenting the contradictory findings, including the specific immunological metrics and their respective supporting references, the system provides necessary context for human experts to weigh the evidence. Together, these results show that an LLM-powered, knowledge graph-driven workflow can refine antigen profiles and support data-informed decisions for tuberculosis vaccine design.

### 3.3 Knowledge-augmented prediction of antigen protective efficacy

**Fig. 3:**
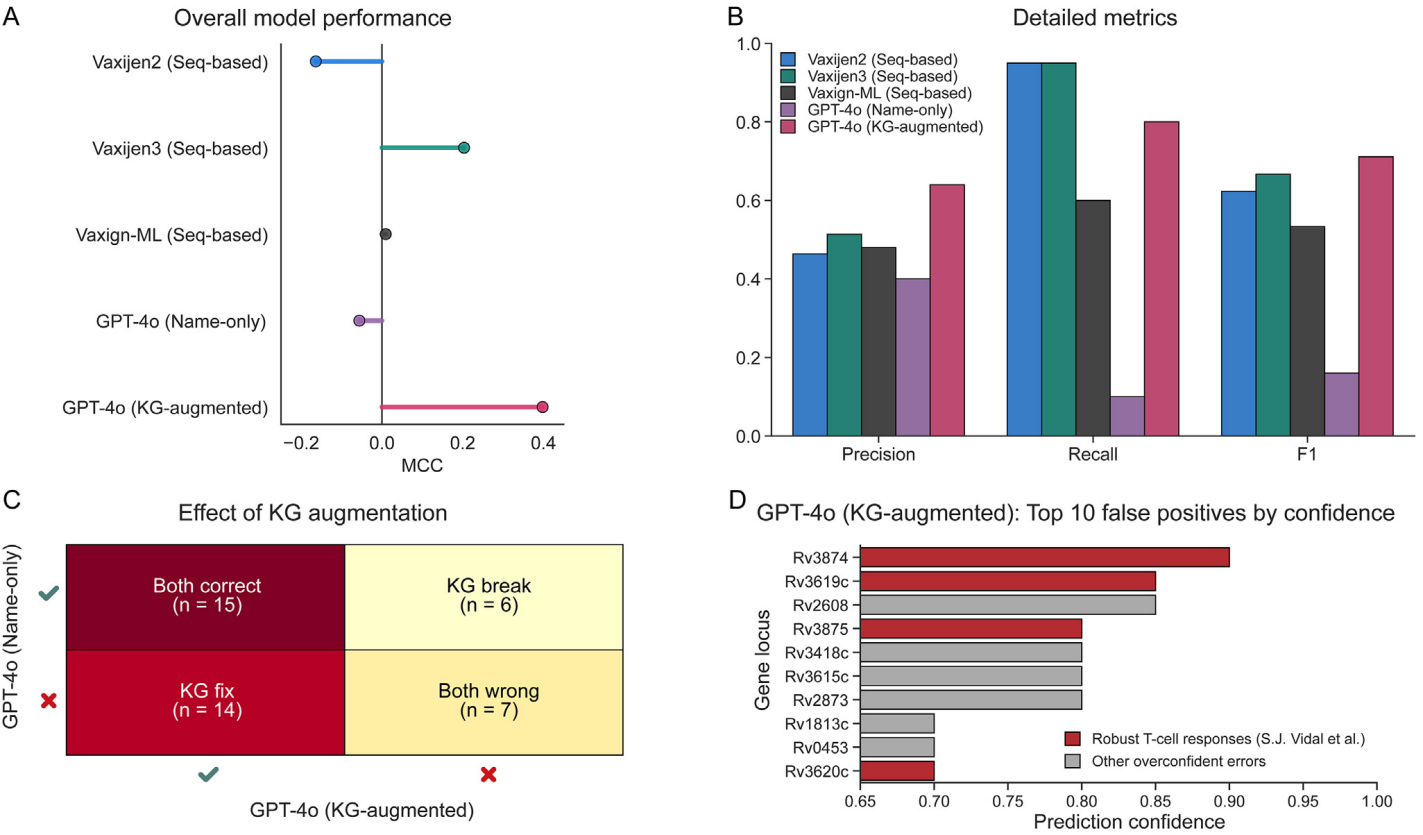
MTB-ImmunogenKG augmentation improves the prediction of antigen protective efficacy. A) and B) Performance comparison of the KG-augmented model, the Name-only control, and Seq-based tools (VaxiJen2, VaxiJen3, Vaxign-ML) on the 42-antigen test set [53]. Metrics shown are (A) MCC as an overall summary metric and (B) Precision, Recall, and F1 score. C) A 2 × 2 matrix quantifying the effect of augmentation. KG fix (n = 14) are antigens misclassified by the Name-only control but corrected by the KG-augmented model. KG break (n = 6) are antigens correctly classified by the control but misclassified by the augmented model, resulting in a net gain of 8 correct classifications. D) Error analysis of the top 10 most confident false positives from the KG-augmented model. Four of these errors (highlighted in red) correspond to antigens known to elicit robust T-cell responses despite limited protective efficacy [53]. Abbreviations: MTB, *Mycobacterium tuberculosis*; MCC, Matthews correlation coefficient.

We tested whether structured evidence in MTB-ImmunogenKG confers predictive value using the evaluation framework detailed in Section 2.5. The KG-augmented model achieved the highest MCC on the evaluation set (Fig. 3A), improving MCC by 0.19 over the best Seq-based tool (Vaxijen3) and by 0.45 over the Name-only control, reaching an MCC of 0.40. The Name-only model performed poorly, driven by very low recall (Fig. 3B). The Seq-based tools showed the opposite tendency, achieving high recall but lower precision. The KG-augmented setting balanced these extremes.

Per-antigen analysis (Fig. 3C) showed that augmentation resulted in 14 KG fix instances and 6 KG break instances, for a net gain of 8 correct classifications. This links the improvement directly to evidence supplied by MTB-ImmunogenKG. Error analysis further illustrates task difficulty (Fig. 3D): among the ten most confident false positives, four antigens (Rv3874, Rv3619c, Rv3875, Rv3620c) belong to a small group reported by S.J. Vidal et al. [53] to elicit robust T-cell responses despite limited protective efficacy.

## 4 Discussion

MTB-ImmunogenKG consolidates sentence-level immunological evidence into a provenance-linked resource. Organized by knowledge patterns for tuberculosis antigen selection, it converts evidence from large-scale literature into a structured basis for antigen profiling and protective efficacy evaluation. By returning traceable summaries that also surface contradictions, the resource facilitates rational antigen prioritization and streamlines the experimental design cycle for next-generation tuberculosis vaccines.

MTB-ImmunogenKG focuses on knowledge-driven, protein-level antigen candidates, and complements existing selection strategies. First, artificial intelligence tools that operate on sequences (e.g., models for protein interaction) mainly nominate sequence-based candidates. Our resource can corroborate and cross-validate these suggestions using literature evidence. Second, peptide-level tools can be applied downstream to propose epitopes from the protein-level results. The same framework can be adapted to other bacterial vaccine programs by redefining knowledge patterns and rebuilding knowledge graphs for the target pathogen.

This work is bounded by literature coverage and by normalization accuracy, and some patterns such as Antigen synergy and Adjuvant remain sparse. These constraints are typical of the field. Even so, MTB-ImmunogenKG organizes dispersed findings into traceable evidence that supports antigen profiling and evaluation of protective efficacy, offering a practical bridge from data retrieval to decision support.
