# Supplementary material for: MTB-ImmunogenKG: An LLM-assisted knowledge graph for antigen selection in tuberculosis vaccine research
Source: Biosaf Health. 2026 Feb 4;8(2):143–9. doi: 10.1016/j.bsheal.2026.02.001 (PMC13147775; doi:10.1016/j.bsheal.2026.02.001)
Supplement: Supplementary Data 1 [file mmc1.docx]

# **Supplementary Figures**

**
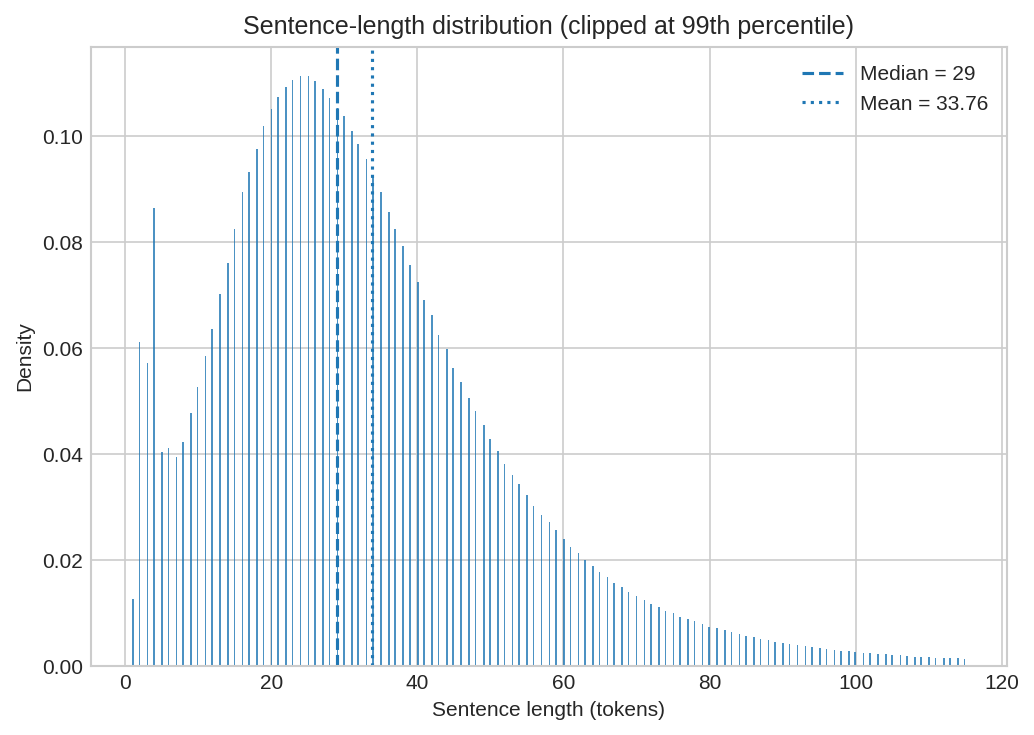
**

**Fig. S1. Sentence-length distribution of the initial corpus (n = 9,310,502).** Vertical lines mark the median and mean.

# **Supplementary Tables**

**Table S1. Entity types, normalization methods, and statistics of extracted mentions and unique entities**

| **No.** | **Entity type** | **Normalization method** | **Unique entities** | **Mentions** |
| --- | --- | --- | --- | --- |
| 1 | Mycobacterium tuberculosis antigen | reference database | 3,154 | 220,816 |
| 2 | Cell type/subtype | LLM-assisted | 266 | 791,973 |
| 3 | Host | LLM-assisted | 7 | 430,979 |
| 4 | Cytokine | reference database | 1,773 | 497,677 |
| 5 | Receptor | reference database | 3,419 | 159,875 |
| 6 | Strain | LLM-assisted | 124 | 296,736 |
| 7 | Type of immune response | LLM-assisted | 4 | 257,377 |
| 8 | Antibody | reference database | 1,300 | 30,602 |
| 9 | Bacterial surface secretion system | LLM-assisted | 267 | 22,206 |
| 10 | Chemokine | reference database | 531 | 40,540 |
| 11 | Adjuvant | reference database | 353 | 36,388 |
| 12 | Complement | reference database | 260 | 4,884 |
| 13 | Major histocompatibility complex | reference database | 561 | 20,988 |
| 14 | Vaccine structural component | LLM-assisted | 38 | 62,537 |
| Totals |  |  | 12,309 | 2,873,578 |

***Notes:*** Entity mentions were identified across all MTB-related texts and then normalized to unique entity records.

# **Supplementary Data Legends**

**Supplementary Data 1 Antigen Profile Result:** It contains contradiction-aware summarization outputs for 17 profiled antigens, referenced in Results 3.2.

**Supplementary Data 2: Knowledge Summaries for Protective Efficacy Prediction.** It contains the final knowledge summaries for the 40 antigens retrieved from MTB-ImmunogenKG, which were used as input for the KG-Augmented model, referenced in Methods 2.5. Two antigens from the evaluation set, Rv2666 and Rv1199c/Rv2512c, were not covered by the KG.

**Supplementary Data 3: Prompt for Protective Efficacy Prediction.** It contains the structured prompt used to instruct the LLM to predict protective efficacy, defining the experimental context and predictive cues, referenced in Methods 2.5.

**Supplementary Data 4: Protective Efficacy Prediction Results.** It contains the complete prediction results for all 42 antigens, comparing the KG-Augmented model against the Name-only model and Seq-Based model, referenced in Methods 2.5.
